# Experimental method to assess depth sensing limits of inelastic scattering measurements using spatial-offset Raman spectroscopy imaging

**DOI:** 10.1117/1.JBO.30.S3.S34108

**Published:** 2025-09-18

**Authors:** Hugo Tavera, Guillaume Sheehy, Patrick Orsini, Jacques Bismuth, Marie-Maude de Denus-Baillargeon, Maroun Massabki, Jean-François Masson, Frederic Leblond

**Affiliations:** a Polytechnique Montreal, Department of Physics Engineering, Montreal, Quebec, Canada; b Centre de Recherche du Centre Hospitalier de l’Université de Montréal, Imaging and Engineering Axis, Montreal, Quebec, Canada; c Optech, Montreal, Quebec, Canada; d Université de Montréal, Institut Courtois, Centre interdisciplinaire de recherche sur le cerveau et l’apprentissage, Quebec Center for Advanced Materials, Regroupement québécois sur les matériaux de pointe, Department of Chemistry, Montreal, Quebec, Canada

**Keywords:** Raman spectroscopy, inelastic scattering, depth-resolved imaging, fluorescence, tissue optics, biochemistry

## Abstract

**Significance:**

The relationship between spatial offset and tissue sensing depth is not well understood in spatial offset Raman spectroscopy (SORS). Detection of the subsurface biochemical composition could improve clinical translation of SORS-based methods, including for lumpectomy margin characterization in breast cancer surgery.

**Aim:**

We aimed at developing an experimental method to establish a relationship between spatial offset in SORS and sampling depth. The technique was developed using a custom hyperspectral line-scanning imaging system optimized for Raman spectroscopy detection.

**Approach:**

Bilayer phantoms were produced with top and bottom layers made of material with different Raman spectroscopy signatures, i.e., poly(dimethylsiloxane) polymer (PDMS) and Nylon. The top layer of PDMS had different values of absorption and reduced elastic scattering coefficients, as well as a thickness up to ∼3  mm. A metric was used, called spectral angle mapper, that allowed for comparing SORS measurements with reference spectra of pure PDMS and Nylon. That metric was used to develop a technique predicting sensing depth for different values of spatial offset. A proof-of-concept study was performed to assess the performance of the method in biological tissue, demonstrating detectability of protein-rich tissue across layers of Intralipid and porcine fat to simulate the optical properties of human adipose tissue.

**Results:**

A total of 60 optical phantoms with varying optical properties and top layer thicknesses were imaged and processed to estimate sampling depth as a function of spatial offset. The study demonstrated the detectability of the underlying Nylon layer across a PDMS layer up to 3 mm in thickness. Similarly, the detectability of protein-rich tissue was demonstrated across layers of Intralipid up to 3 mm thick and <2  mm for porcine fat.

**Conclusions:**

We showed the feasibility of using bilayer solid optical phantoms to create correlation curves between the optimal spatial offset for a desired probed depth given the optical properties of the top layer. The technique could facilitate the clinical translation of SORS measurements for tumor detection and margins assessment.

## 1 Introduction

Spontaneous Raman spectroscopy is a well-established imaging method for tissue characterization, due to its molecular specificity and high sensitivity to biochemical content.^1^ A large range of *in vivo* studies were conducted demonstrating the ability of the technique to detect tumor tissue in the context of oncology applications, including cervical, oral, and skin cancer.^2^ We have developed and integrated into the neurosurgical workflow, intraoperative Raman spectroscopy, or iRS. The technique is based on a single-point macroscopic Raman spectroscopy probe system that can detect tumor tissue live, *in situ* during surgical procedures.^3^^–^^5^ We subsequently developed the next-generation system, a hyperspectral macroscopic line-scanning instrument combining Raman spectroscopy and visible imaging to create 2D maps discriminating tumor from normal tissue from machine learning models trained using data acquired with the iRS system.^6^^,^^7^ The imaging system was designed for surgical-guidance applications in neurosurgery^8^ and for lumpectomy specimen margins inspection in breast-conserving surgery.^9^

Spatial-offset Raman spectroscopy (SORS) is a method developed to probe materials noninvasively by detecting inelastically scattered photons at depth.^10^^–^^14^ When applied to tissue imaging, the technique can be used to retrieve Raman scattering information from subsurface layers, thereby potentially allowing for probing normal or tumor tissue underneath layers, e.g., blood pooling or tissue scarring. The method relies on the transverse-biased motion of diffused photons in a turbid medium. In its most common embodiment, based on fiber-optics probe systems, inelastically scattered and endogenous fluorescence photons are retrieved by offsetting the collection and excitation fibers by a distance Δs. Depth sensing modulation can then be achieved by varying Δs, with larger values leading to increased tissue penetration.^15^ This, however, poses a conundrum because larger offsets lead to diminished levels of light, further increasing the difficulty of detecting Raman photons superposed with large endogenous fluorescence baselines. In SORS, the illumination fibers are usually at the center of the probe instrument and detection is achieved using optical fibers disposed over the perimeter. Several instruments were developed based on variations of the principle of depth-resolved Raman spectroscopy. This included inverse SORS, where tissue interrogation was achieved from an excitation ring of fibers circumferentially distributed around a collection fiber in the center.^16^ This provided improved light sensitivity, higher permissible exposure of laser intensity, and enhanced flexibility to control spatial offset. Other studies used SORS in combination with surface-enhanced Raman spectroscopy (SERS) with the objective of increasing the sensitivity of subsurface layers using metallic nanoparticles.^17^ The resulting surface-enhanced spatial-offset Raman spectroscopy technique, christened SESORS, was also used to monitor the temperature of nanoparticles during thermal treatment.^18^

Spatial-offset Raman spectroscopy was applied to counterfeit drugs or explosive detection through unopened plastic containers as well as in biomedical applications including the study of bone and joint diseases^19^^–^^22^ or transfusion medium quality assessment.^23^^,^^24^ The technique was also used in the scope of studies in oncology, notably for prostate cancer,^25^ skin cancer,^26^ and breast cancer.^13^^,^^27^^,^^28^ Studies were conducted using Monte Carlo light transport simulations to optimize SORS detection geometries and predict ideal spatial offset values when targeting specific depths.^29^^,^^30^ Later studies used advanced Monte Carlo simulation techniques to provide a more accurate relationship between spatial offset and probed depth, for optical properties consistent with biological tissues.^31^^,^^32^ Our group subsequently developed a versatile Monte Carlo light transport simulation tool that was validated from experimental data and used to predict tissue sampling depth for the iRS system as well as for different scenarios involving spatial offset variations.^33^

An aspect of our hyperspectral macroscopic Raman spectroscopy line-scanning instrument that was not exploited in past studies was its ability to automatically control the spatial offset Δs between the excitation and the detection line. This will be used in this manuscript to present the development of a method relying on bilayer tissue phantoms to predict inelastic scattering depth sensing. The ability of the line-scanning technique will also be evaluated for its potential to reliably retrieve Raman spectroscopy information from subsurface layers. Specifically, this manuscript describes a protocol allowing the fabrication of bilayer optical phantoms and presents an experimental protocol allowing SORS data to be acquired with variable spatial offset values. A technique is presented allowing for estimation of depth sensing based on an experimental method using a quantitative metric computing the relative contribution of different Raman-active materials associated with a spectroscopic measurement. A proof-of-concept experiment is also provided based on more realistic tissue phantoms made from a fat-rich layer disposed over muscle tissue. The latter phantom design was chosen to emulate a situation where tumor tissue needs to be detected through a layer of fat in the context, e.g., of breast-conserving surgery procedures.

## 2 Materials and Methods

### 2.1 Tissue Phantoms

Bilayer phantoms were produced from two Raman-active materials, Nylon and poly(dimethylsiloxane) polymer (PDMS), to provide an experimental framework allowing for the estimation of SORS depth sampling using a custom line-scanning Raman spectroscopy system for different spatial offset values. The phantoms were fabricated using a Nylon disc (bottom layer) on which a semi-infinite layer and PDMS was superposed (top layer). Nylon discs were 9.9±0.5  mm thick with a 5-cm diameter. For the PDMS layer, a SYLGARD 184 silicon elastomer base was used, mixed with a curing agent at a 10:1 (base/curing agent) weight ratio.^34^ The optical properties of the PDMS layer were varied by modifying the concentration of absorption and elastic scattering agents. The absorption agent was black Indian Ink mixed with an ethanol-based stock solution at a concentration of 48  mg/mL. The scattering agent was titanium dioxide (${\mathrm{TiO}}_{2}$) powder mixed in a similar ethanol-based stock solution at a concentration of 42  mg/mL. Titanium dioxide is frequently employed in biomedical optics research as a scattering medium to simulate light diffusion in biological tissues.^35^^–^^39^

In total, 10 different recipes of PDMS were produced. The volume of the stock solution added to PDMS was calculated to obtain the concentrations listed in Table 1. For each PDMS recipe, six solid phantoms were made associated with different thicknesses ranging from 0.5 to 3 mm by 0.5-mm increments. This resulted in 60 optical phantoms associated with different values of top layer thickness and optical properties (absorption, elastic scattering). The phantoms were initially placed in a vacuum chamber for 1 h to eliminate all bubbles introduced when mixing the PDMS recipes. They were then placed in an oven at 80°C for 2 h for curing. For each recipe, an extra slab of PDMS—without the bottom layer of Nylon—was produced and used to compute the absorption and reduced elastic scattering coefficients using a spectrophotometer (model: Lambda 1050 UV/Vis/NIR, PerkinElmers). The total detected reflectance and total detected transmission were measured with the instrument between 400 and 1000 nm by 10 nm increments. The coefficients $\mu_{a}$ and $\mu_{s}^{\prime }$ were then computed using the Inverse Adding-Doubling (IAD) software.^40^

**Table 1:** Concentrations of absorption and scattering agents mixed with PDMS for the ten different optical phantom recipes. Letters A or D in the recipe name referred to the presence of either an absorbing or a diffusive agent, respectively. An X meant no agent was used.

Recipe name	Concentrations of absorption and scattering agents mixed with PDMS (mg/ml)	
	Indian ink	${\mathrm{TiO}}_{2}$
C01AX	1.0	0.0
C02XD	0.0	1.0
C03AD	1.0	1.0
C04AD	1.0	0.5
C05AD	0.5	1.0
C06AD	0.5	0.5
C07AX	0.5	0.0
C08XD	0.0	0.5
C09AD	1.0	5.0
C10AD	1.0	10.0

### 2.2 Imaging System

Measurements were made using a macroscopic line-scanning hyperspectral imaging system optimized for inelastic scattering detection for which details were provided elsewhere.^6^^,^^7^ Briefly, the excitation branch of the imaging system was composed of a laser centered at 785 nm providing a line-shaped excitation beam and a white light source with wide field illumination between 400 and 700 nm. Laser and white light illumination were conveyed through an excitation fiber bundle to an imaging probe and onto the sample. The re-emitted light was collected back by the imaging probe and a collection fibre bundle. A dichroic mirror was used to transfer a fraction of the light to a white light camera. The other fraction of the light was conveyed to a hyperspectral camera across a high-pass filter limiting excitation light bleed-through. The illumination laser line and the detection line were scanned either independently or synchronously, at any desired spatial offset Δs. The spectroscopic, red-shifted signal was conveyed to an imaging spectrometer to produce hyperspectral images composed of inelastically scattered photons (Raman scattering) over a baseline associated with endogenous fluorescence. The white light images of the sample were captured using the white-light color camera. The spatial resolution of the hyperspectral images was ∼250  μm with a spectral resolution of $6\;{\mathrm{cm}}^{-1}$. The Raman spectra were acquired in the fingerprint region from 400 to $2100\;{\mathrm{cm}}^{-1}$. The measurements were made without contact, at a working distance of 40 mm. The laser line and the detection line had a 400  μm width and 1 cm length at the level of the sample. The field-of-view of the system was $1\;cm^{2}$. The system was controlled by custom software that allowed acquisition parameters to be set by the user, including laser power, exposure time per line, and number of repeated measurements.

### 2.3 Imaging Protocol

**Fig. 1:**
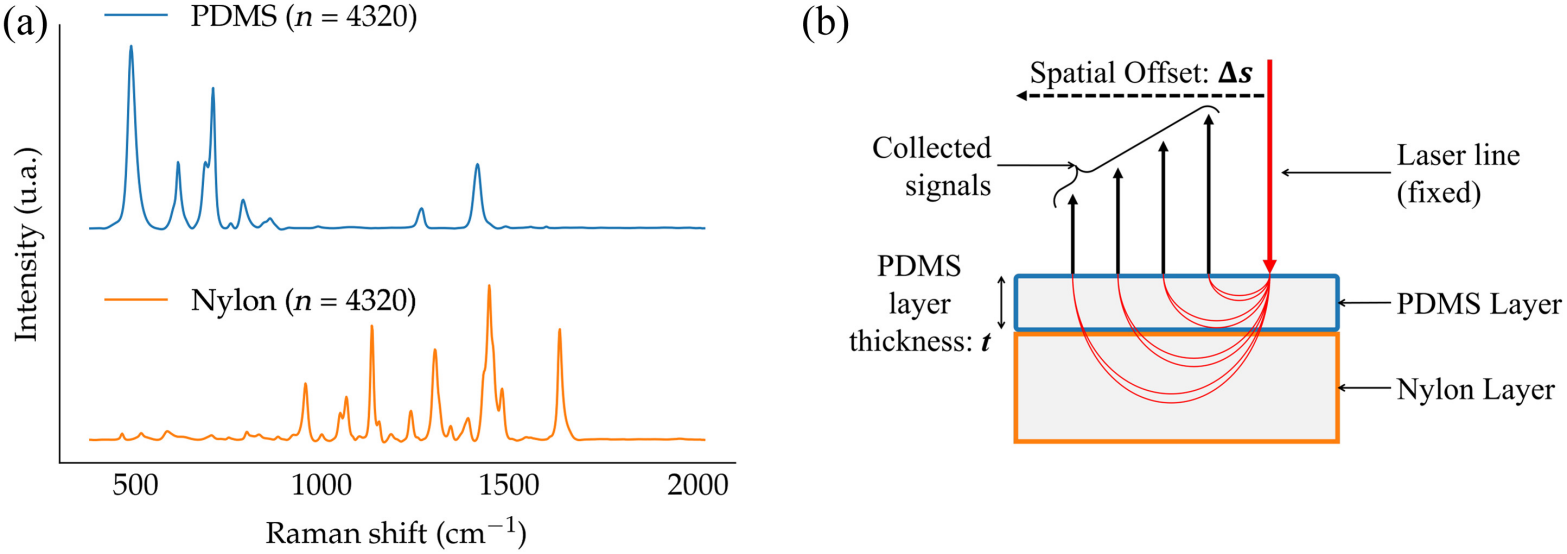
Schematic depiction of the experimental protocol used to image the two-layer PDMS/Nylon optical phantoms: (a) spectra of pure PDMS (blue/top) and Nylon (orange/bottom) obtained by averaging n measurements acquired with the line-scanning system with a spatial offset Δs=0  mm; (b) SORS imaging protocol associated with a fixed laser line and a detection line scanned at different offsets values Δs from 0 to 10 mm by 0.25 mm increments. The imaging protocol was repeated for all 60 optical phantoms, each with different layer thicknesses t and optical properties (absorption and elastic scattering).

Figure 1(a) shows PDMS (top/blue) and Nylon (bottom/orange) Raman spectra obtained by averaging measurements from three Raman images of pure phantoms for an offset Δs=0  mm. Figure 1(b) shows a schematic of the imaging protocol of the optical phantoms, including a conceptual depiction of the path of higher probability followed by photons collected from spatial offset measurements. SORS hyperspectral images were acquired by fixing the laser line on one side of the square field-of-view of the system, and the surface was scanned by the detection line. The scanned line started at the same position as the laser line (Δs=0  mm) with subsequent measurements acquired at larger spatial offsets Δs by 250  μm increments, totaling 40 measurements per phantom. The imaging spectrometer acquired one Raman image for each of the 40 positions of the scanning line. Each of those images had 1024×42  pixels. The larger dimension (1024 pixels wide) consisted of the wavenumber shift values associated with the x-axis of 42 Raman spectra, each with a different position along the scan line. Each image of a line was acquired with an exposure time of 20 s. The scanning direction (perpendicular to the scan line) corresponded to increasing spatial offset values Δs between the laser line and the scanning line. The resulting hyperspectral image was of dimension 1024×42×40, i.e., a wavenumber shift dimension and two spatial dimensions. The square field of view was positioned in the center of the optical phantoms to eliminate edge effects. This protocol was repeated for each of the 60 optical phantoms described in Sec. 2.1.

Two different calibration measurements were made for (1) Raman shift calibration on the spectrometer and (2) system response and illumination uniformity correction. Acetaminophen Raman imaging served as a wavenumber shift standard for calibrating the Raman shifts associated with pixels on the CCD sensor of the spectrometer. An acetaminophen calibration image was acquired as the average of a series of three images at 500 ms per line (total: 20 s). System response correction was done by imaging a relative intensity correction standard developed for Raman Spectroscopy at 785-nm excitation from the National Institute of Standards and Technology (NIST), reference material number 2241. The NIST standard calibration image corresponded to the average of a series of four images at 5 s per line (total: 200 s).

### 2.4 Data Processing

#### 2.4.1 Spectral pre-processing

Processing was done using Python 3.12 with common libraries, including Numpy, Pandas, and SciPy, as well as the custom open-sourced code, the Open Raman Processing Library (ORPL).^41^ One SORS image was composed of 40 Raman images, stored as raw TIFF files. The TIFF files stored information from 1024 pixels (wavenumber axis) × 42 pixels (spatial dimension transverse to the scan line). The first 120 pixels along the wavenumber axis were truncated out of the image files as they correspond to the wavelength range below the high-pass filter cutoff. Cosmic rays were automatically identified and removed using tools available in ORPL. The acetaminophen calibration image was then used to correct for the x-axis curvature of the Raman bands on the spectrometer CCD sensor, known as “smile” spectral distortions. This calibration frame was also used to correlate pixel numbers to wavenumber values based on the known locations of the acetaminophen Raman bands. The NIST standard calibration image was used for the system response and illumination uniformity corrections. Intrinsic fluorescence from the optical phantoms was then removed using the *Bubblefill* algorithm from ORPL. Finally, standard normal variate (SNV) normalization was applied to each of the 42 Raman spectra composed of 904 wavenumber values.

#### 2.4.2 Spectral angle measurement (SAM)

A metric was implemented that quantified the relationship between the spatial offset Δs and the ability of the system to detect subsurface layers of Raman-active material.^39^ The method relied on the natural hypothesis that measured Raman spectra, from the bilayer phantoms, were a linear combination of a PDMS spectrum and a Nylon spectrum. The metric quantified the similarity of any measured Raman spectrum with either of the two basis spectra, i.e., PDMS or Nylon [Fig. 1(a)]. This was achieved using the Spectral Angle Metric (SAM) that computed the higher-dimensional angle between two Raman spectra based on the scalar product between two N-dimensional vectors (N=904), in wavenumber space

$$\mathrm{SAM}(\overset{\to }{R},\overset{\to }{S})=\frac{\overset{\to }{R}\cdot \overset{\to }{S}}{\left|\overset{\to }{R}|\cdot |\overset{\to }{S}\right|}=\frac{\sum R_{i}S_{i}}{\sqrt{\sum R_{i}^{2}}\sqrt{\sum S_{i}^{2}}}.$$ (1)

In the SAM formula, $\vec{S}$ is the measured spectrum, whereas $\vec{R}$ is the reference spectrum, i.e., PDMS or Nylon. Using this definition, the SAM ranged from −1 to 1, where a value of +1 indicated spectral features were identical to the reference spectrum (i.e., the spectra were colinear and “point” in the same direction) and a value of 0 indicated no correspondence between the two spectra (i.e., they were linearly independent). The SAM was computed for all Raman spectra from the SORS images of all bilayer phantoms, for both PDMS and Nylon as reference spectra. This provided SAM value curves, for PDMS (${\mathrm{SAM}}_{\mathrm{PDMS}}$) and Nylon (${\mathrm{SAM}}_{\text{Nylon}}$), as a function of the spatial offset Δs for all sets of $\mu_{a}$ and $\mu_{s}^{\prime }$ values associated with the top layer made of PDMS.

The naïve expectation was that, for any spatial offset Δs, ${\mathrm{SAM}}_{\mathrm{PDMS}}=1$ and ${\mathrm{SAM}}_{\text{Nylon}}=0$ for a pure PDMS phantom, and vice versa for a pure Nylon phantom. However, as Δs increases the overall photonic signal-to-noise ratio (SNR) will decrease, eventually leading to the absence of detectable Raman features of PDMS or Nylon. Spectral features should then be detected that are associated with the fluorescence background of the phantom (no Raman features remaining), either from stochastic noise or residual effects of the spectral response of the instrument. As a result, a decreasing value of SAM—as a function of spatial offset—should be interpreted as a loss of detectability for a given material type, but it will never reach a value of zero, even for large values of Δs. We hypothesize that the evolution of the ${\mathrm{SAM}}_{\mathrm{PDMS}}$ and ${\mathrm{SAM}}_{\text{Nylon}}$ metrics as a function of Δs can be used as a mean to assess inelastic scattering sensing depth. In fact, as the spatial offset increases, the major contributor to the measured spectrum is expected to shift from PDMS (top layer) to Nylon (bottom layer) with their SAM values decreasing and increasing, respectively.

### 2.5 Proof of Concept: Bilayer Biological Phantoms

**Fig. 2:**
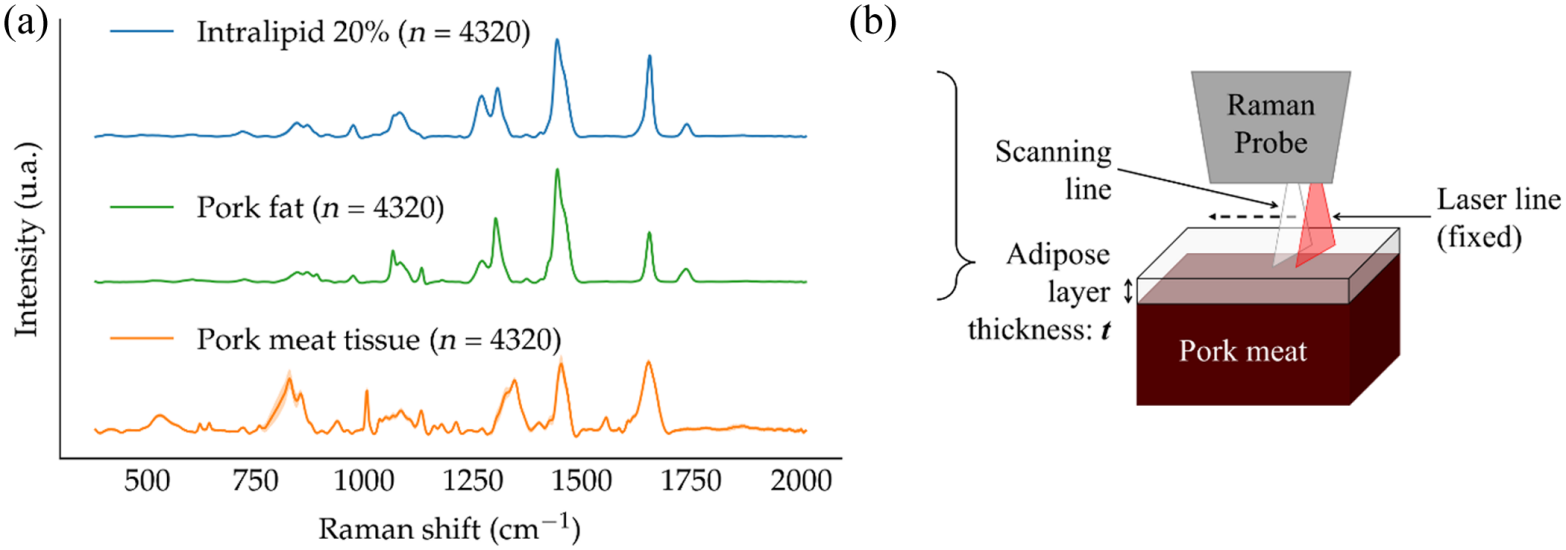
Proof-of-concept imaging protocol: (a) average and standard deviation of pure spectrum of Intralipid (top/blue), pork fat tissue (middle/green), and pork muscle tissue (orange/bottom); (b) imaging protocol where the laser line is fixed, and the scanning line is progressively moved away from the laser line. The muscle cube is either immersed in Intralipid, or a slice of fat is put on top of it. The Intralipid layer thickness is measured by the difference in height of the two media. The porcine fat slices were excised from the same piece of meat used in the experiment.

A proof-of-concept experiment was designed to assess the performance of the system in samples more closely resembling biological tissues. A bilayer sample made of a muscle tissue (pork meat purchased in a grocery store) underlayer, and a top layer of fat substitute (Intralipid 20% emulsion) provided a configuration with Raman optical properties, e.g., a situation that could be encountered in fatty human tissue (Fig. 2). The optical properties of the Intralipid solution were estimated to be $\mu_{a}=0.03\;{\mathrm{cm}}^{-1}$ and $\mu_{s}^{\prime }=200\;{\mathrm{cm}}^{-1}$.^42^ Pure spectra were initially acquired for each medium, i.e., meat and Intralipid (Fig. 2). Intralipid and muscle tissue shared common spectral features but differed in several spectral regions, including the aromatic amino acid peak associated with proteins around $1004\;{\mathrm{cm}}^{-1}$. The experimental setup is schematized in Fig. 2, where a cube of pork meat was placed in a transparent container and Intralipid was poured over it. SORS images were acquired of tissue phantoms with Intralipid thicknesses t of 0.5, 1, and 3 mm. The imaging protocol was described in Sec. 2.3. The SAM values were computed for meat (${\mathrm{SAM}}_{\text{Meat}}$) and Intralipid (${\mathrm{SAM}}_{\mathrm{IL}}$), and they were plotted as a function Δs as described in Sec. 2.4.2 for the PDMS/Nylon bilayer phantoms. The same experimental protocol was applied in a context that more closely mimicked the presence of an adipose tissue layer situated between the imaging system and underlying protein-rich tissue. To achieve this, the Intralipid layers were replaced with a layer of pure adipose tissue (t=1, 3, and 6 mm) excised from a piece of porcine meat used in the initial experiment. This substitution was performed to more closely mimic real human tissue, as the optical absorption properties of the adipose tissue layer more accurately reflected those of human adipose tissue when compared with Intralipid.

## 3 Results

### 3.1 Bilayer PDMS/Nylon Phantoms

Following the protocol described in Sec. 2.1, a set of 60 optical phantoms was created, made of 10 different PDMS recipes leading to 10 optical property pairs, with six different PDMS layer thicknesses per PDMS recipe, ranging from 0.37 to 3.06 mm. Table 2 lists all measured thicknesses for the PDMS layer in all 60 optical phantoms, alongside the optical properties of each PDMS recipe, calculated with the IAD software. Absorption coefficient $\mu_{a}$ ranged from $0.09\;{\mathrm{cm}}^{-1}$ (no absorbing agent) to $8.15\;{\mathrm{cm}}^{-1}$, while scattering coefficient $\mu_{s}^{\prime }$ ranges from $0.02\;{\mathrm{cm}}^{-1}$ (no scattering agent) to $78.13\;{\mathrm{cm}}^{-1}$, with most values distributed between 0.02 and $8.08\;{\mathrm{cm}}^{-1}$. Absorption and scattering values were nonzero despite the absence of added agents because of the intrinsic optical properties of PDMS.

**Table 2:** List of all the PDMS layer thicknesses for each PDMS recipe. Letters A, D, or X in the recipe name refer to the presence of absorbing agent (A), diffusive agent (D), or if the respective agent is not present (X). PDMS layer thickness #1 is not shown because it was only used to measure optical properties and is not a dual-layer optical phantom. Absorption coefficient $\mu_{a}$ and reduced scattering coefficient $\mu_{s}^{\prime }$ are evaluated at 785 nm, as the mean value between the values at 780 and 790 nm calculated by IAD software.

Recipe name	PDMS layer thicknesses t per recipe (μm)						$\mu_{a}$ (${\mathrm{cm}}^{-1}$)	$\mu_{s}^{\prime }$ (${\mathrm{cm}}^{-1}$)
	#2	#3	#4	#5	#6	#7		
C01AX	370	810	1190	1700	2120	2640	2.35	0.06
C02XD	430	600	1070	1650	2030	2500	0.09	6.94
C03AD	450	840	990	1840	2320	2780	5.78	6.21
C04AD	720	910	1510	1900	2360	3060	3.66	1.89
C05AD	550	920	1220	1580	2290	2710	2.83	8.08
C06AD	530	930	1360	1800	2290	2600	2.05	3.64
C07AX	430	950	1790	2170	2700	3170	2.08	0.02
C08XD	650	970	1640	1980	2470	2810	0.18	3.74
C09AD	490	800	1190	1560	1900	2480	8.15	47.61
C10AD	480	760	1090	1620	1990	2410	7.04	78.13

**Fig. 3:**
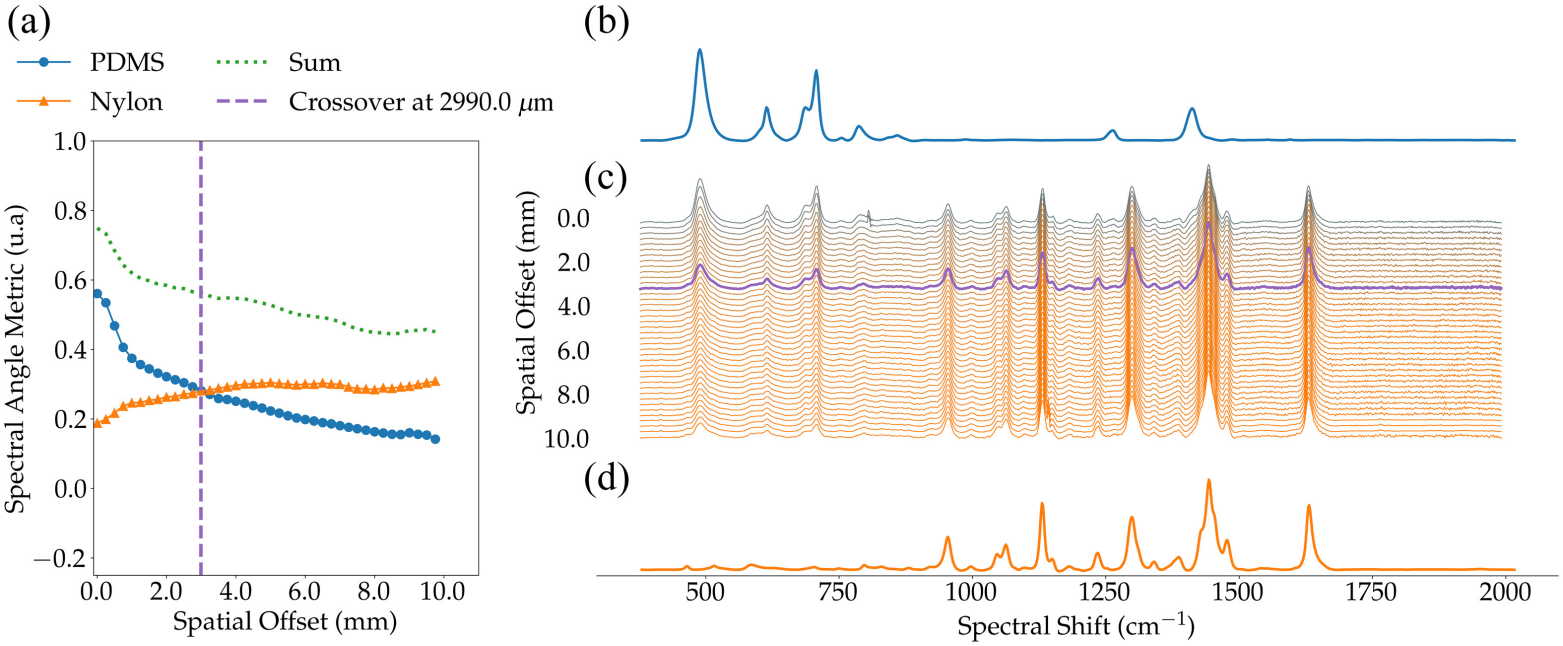
Spectral Angle Mapper (SAM) metric evaluated for PDMS and Nylon spectra for a representative optical phantom: C02XD5 (t=1650  μm, $\mu_{a}=0.09\;{\mathrm{cm}}^{-1}$, $\mu_{s}^{\prime }=6.94\;{\mathrm{cm}}^{-1}$): (a) evolution of ${\mathrm{SAM}}_{\mathrm{PDMS}}$ (blue/circle) and ${\mathrm{SAM}}_{\text{Nylon}}$ (orange/triangle) as well as the sum (green/dotted) of both SAM when increasing the spatial offset and the SAM crossover offset labeled $\Delta s_{\mathrm{CO}}$ (vertical dashed line); (b) pure spectrum of PDMS; (c) evolution of the measured spectrum when increasing spatial offset Δs, which was used to calculate the SAM (a). The purple trace highlights the spectrum at $\Delta s_{\mathrm{CO}}$, i.e., the SAM crossover point; and (d) pure spectrum of Nylon.

The data processing protocol described in Sec. 2.4 was applied to all bilayer phantoms, and the SAM values (${\mathrm{SAM}}_{\mathrm{PDMS}}$, ${\mathrm{SAM}}_{\text{Nylon}}$) were computed for all Δs=0  mm and Δs≠0  mm spectra. Figure 3 showed the results for phantom C02XD5, which had parameters t=1.650  mm, $\mu_{a}=0.09\;{\mathrm{cm}}^{-1}$, and $\mu_{s}^{\prime }=6.94\;{\mathrm{cm}}^{-1}$ (Table 3). Similar results were obtained for all phantoms. Figure 3(a) shows the evolution of the SAM when increasing the spatial offset, and Fig. 3(c) shows processed Raman spectra averaged for all spatial offset values. Figures 3(b) and 3(d) showed the PDMS and Nylon spectra, respectively. The blue (circles) line showed the ${\mathrm{SAM}}_{\mathrm{PDMS}}$ as a function of Δs, indicating that the similarity of the measurement with a pure PDMS spectrum decreased for larger spatial offset values. The trend was reversed for ${\mathrm{SAM}}_{\text{Nylon}}$. In fact, the orange (triangles) line showed an increase of ${\mathrm{SAM}}_{\text{Nylon}}$ with Δs, indicating an improved correspondence with Nylon as the spatial offset increased. These trends are consistent with an increased sensing depth for larger spatial offsets.

**Table 3:** List of the recipes used to achieve different optical properties (absorption, reduced elastic scattering) for the PDMS layer with corresponding parameters A and φ of the fitted curves in Fig. 4.

Recipe name	$\mu_{a}$ (${\mathrm{cm}}^{-1}$)	$\mu_{s}^{\prime }$ (${\mathrm{cm}}^{-1}$)	Fitted parameters	
			A	φ
C01AX	2.35	0.06	355	0.286
C02XD	0.09	6.94	198	0.259
C03AD	5.78	6.21	47	0.475
C04AD	3.66	1.89	128	0.396
C05AD	2.83	8.08	79	0.404
C06AD	2.05	3.64	152	0.401
C07AX	2.08	0.02	48	0.663
C08XD	0.18	3.74	825	0.166
C09AD	8.15	47.61	1	0.880
C10AD	7.04	78.13	1	0.864

The green (dotted) line in Fig. 3(a) showed the sum of the SAM values, i.e., ${\mathrm{SAM}}_{\text{Total}}={\mathrm{SAM}}_{\mathrm{PDMS}}+{\mathrm{SAM}}_{\text{Nylon}}$, as a function of spatial offset. As expected, the overall SAM value was high for Δs=0  mm and decreased monotonically as the spatial offset increased. The ${\mathrm{SAM}}_{\text{Total}}$ value decreased up to a point where the Raman spectrum neither showed the spectral features of PDMS or Nylon. As a rule of thumb, loss of detectability of the Raman-active materials occurred when both ${\mathrm{SAM}}_{\mathrm{PDMS}}$ and ${\mathrm{SAM}}_{\text{Nylon}}$ started to decrease (as a function of Δs), i.e., at the turnover point where the SAM associated with the compound of the bottom layer plateaued out.

### 3.2 Estimated Penetration Depth in Synthetic Bilayer Phantoms

The vertical purple (dashed) line in Fig. 3(a) identifies a critical point in the SAM versus Δs curves, i.e., the spatial offset value where ${\mathrm{SAM}}_{\mathrm{PDMS}}$ and ${\mathrm{SAM}}_{\text{Nylon}}$ are intercepted. That point always occurred, for all phantoms, shortly before the plateauing point for ${\mathrm{SAM}}_{\text{Nylon}}$, in terms of Δs value. Physically, the crossover point corresponded to the spatial offset at which both PDMS and Nylon signals contributed approximately equally to the measured spectrum.

**Fig. 4:**
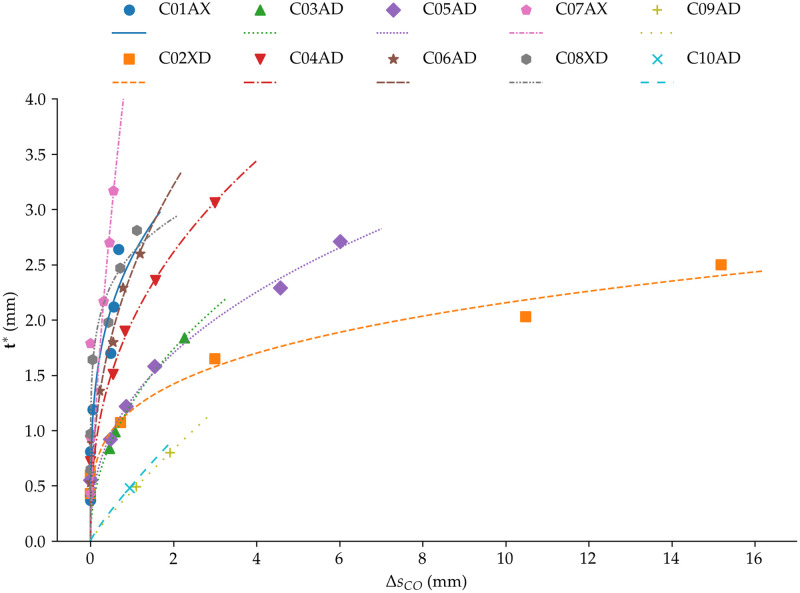
Estimated depth penetration ($t^{*}$) in relation to the spatial offset at the SAM crossover ($\Delta s_{\mathrm{CO}}$) for each PDMS recipe. Each sequence of data points represents the relation between the spatial offset and the corresponding probed depth, which depends on the optical properties of the top PDMS layer. Each curve was fitted with Eq. (2) to highlight trends.

Each phantom, with a given PDMS thickness t and specific optical properties $\mu_{a}$ and $\mu_{s}^{\prime }$, has one characteristic crossover point value $\Delta s_{\mathrm{CO}}(t,\mu_{a},\mu_{s}^{\prime })$. The physical interpretation of this parameter is that to probe a depth of t, the spatial offset of the system Δs needs to be set at the crossover point value $\Delta s_{\mathrm{CO}}$. This meant that the system could then achieve detectability of the bottom layer material (Nylon). Thus, the corresponding value of t can effectively be used as a measure of the sensing depth of the imaging system for optical properties $\mu_{a}$ and $\mu_{s}^{\prime }$ when the distance between the source and detection lines is $\Delta s_{\mathrm{CO}}$. Figure 4 shows the data points associated with PDMS thickness t (y-axis) as a function of $\Delta s_{\mathrm{CO}}$ (x-axis) for all phantoms in Table 2. In the figure, different colors and symbols represent phantoms with the same optical properties $\mu_{a}$ and $\mu_{s}^{\prime }$ of the PDMS top layer.

The data points in Fig. 4 could be used as the basis for the development of a model predicting depth sensing from spatial offset for a given set of optical properties $\mu_{a}$ and $\mu_{s}^{\prime }$. To this end, a curve was fitted for each set of optical properties with the following form:

$$t^{*}=A\cdot \Delta s_{\mathrm{CO}}^{\varphi },$$ (2)

where $t^{*}$ is the estimated depth penetration and $\Delta s_{\mathrm{CO}}$ is the spatial offset of the SAM crossover point. The fitting parameters φ and A depend on the optical properties of the PDMS top layer. The fitted parameters for each PDMS recipe are presented in Table 3, alongside their respective optical properties. The parameter $t^{*}$ corresponds to predictions computed from Eq. (2) based on values of the experimental parameter t associated with each of the phantoms in Table 2.

Inspection of the different curves in Fig. 4 revealed a strong dependence of depth sensitivity with regard to spatial offset when the reduced scattering coefficient is weak. For example, in the case of phantoms C07AX and C01AX, which have low scattering ($\mu_{s}^{\prime }<0.07\;{\mathrm{cm}}^{-1}$) for a relatively high absorption ($\mu_{a}=2$ to $3\;{\mathrm{cm}}^{-1}$), the value of spatial offset $\Delta s_{\mathrm{CO}}$ necessary to reach up to a few millimeters of effective depth penetration remained below 0.5 mm. Therefore, even a small variation of the spatial offset increased probing depth sufficiently to detect the bottom Nylon layer. A different physical behavior was observed for phantoms with relatively high elastic scattering ($\mu_{s}^{\prime }=7$ to $10\;{\mathrm{cm}}^{-1}$), such as C02XD ($\mu_{a}=0.09\;{\mathrm{cm}}^{-1}$) and C05AD ($\mu_{a}=2.83\;{\mathrm{cm}}^{-1}$). For CO2XD, the depth sensitivity curve is as would be expected in the presence of higher diffusion. That is, a more gradual increase of the spatial offset crossover point for thicker PDMS layers. For high scattering coefficients, including C09AD ($\mu_{s}^{\prime }∼50\;{\mathrm{cm}}^{-1}$) or C10AD ($\mu_{s}^{\prime }∼80\;{\mathrm{cm}}^{-1}$), diffusion is so strong that the PDMS becomes effectively opaque, and photons are lost without ever reaching the deeper Nylon layer. As a result, no signal from the Nylon sublayer was collected, and the crossover point loses its physical interpretation. In those cases, the measured signal transitioned from PDMS to stochastic noise as the spatial offset increased.

### 3.3 Depth Sensing Proof-of-Principle Study in Bilayer Biological Phantoms

**Fig. 5:**
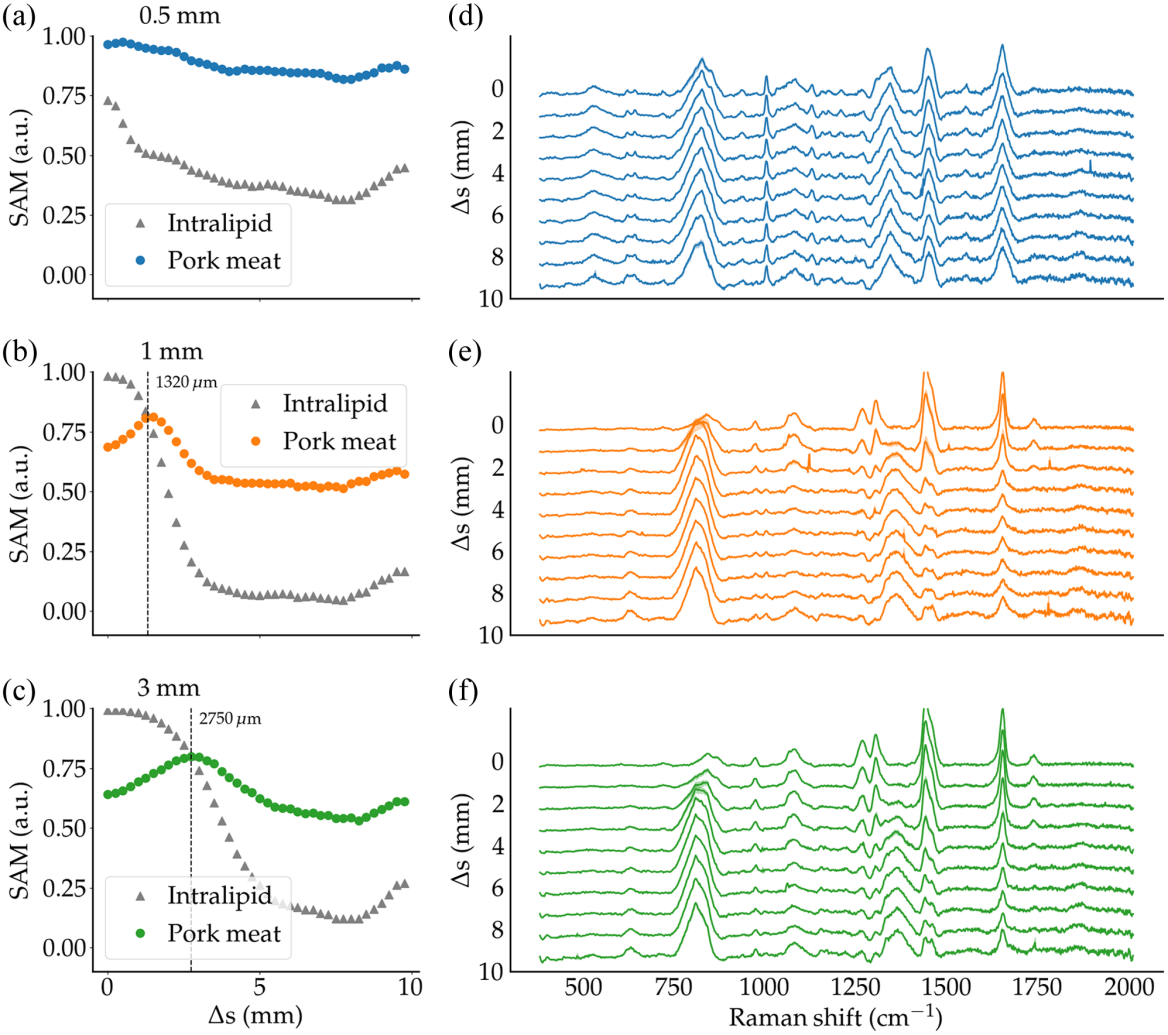
SAM metric evaluated for Intralipid (triangles) and pork meat (circles) for: (a) 0.5-mm Intralipid layer thickness; (b) 1-mm Intralipid layer thickness; and (c) 3-mm Intralipid layer thickness. The crossover offset $\Delta s_{\mathrm{CO}}$ is shown by a vertical dashed line when it occurs. Panels (d), (e), and (f) show the corresponding measured Raman spectra for different values of the spatial offset Δs.

**Fig. 6:**
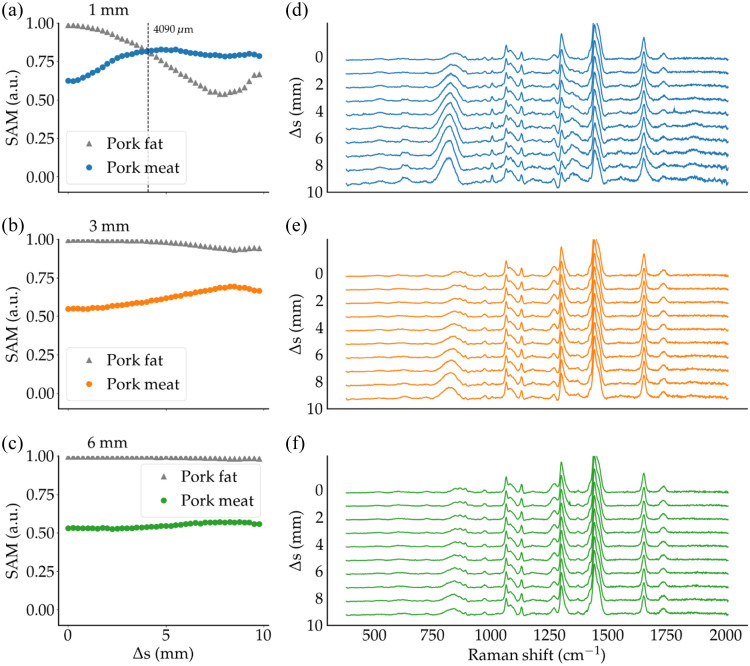
SAM metric evaluated for porcine fat (triangles) and pork meat (circles) for (a) 1-mm fat layer thickness, (b) 3-mm fat layer thickness, and (c) 6-mm fat layer thickness. The crossover offset $\Delta s_{\mathrm{CO}}$ is shown by a vertical dashed line when it occurs. Panels (d), (e), and (f) show the corresponding measured Raman spectra for different values of the spatial offset Δs.

The SAM calculations for pork meat (${\mathrm{SAM}}_{\text{Meat}}$), Intralipid (${\mathrm{SAM}}_{\mathrm{Il}}$), and porcine fat (${\mathrm{SAM}}_{\text{Fat}}$) were performed for the proof-of-concept experiment based on the meat/Intralipid and meat/porcine fat bilayer phantoms. The SAM data as a function of Δs were shown for Intralipid thicknesses of 0.5 mm [Fig. 5(a)], 1 mm [Fig. 5(b)], and 3 mm [Fig. 5(c)]. The data were also shown for porcine fat thicknesses of 1 mm [Fig. 6(a)], 3 mm [Fig. 6(b)], and 6 mm [Fig. 6(c)].

For the Intralipid layers associated with t=1  mm and t=3  mm, ${\mathrm{SAM}}_{\text{Meat}}$ typically increased from Δs=0  mm up to the crossover point $\Delta s_{\mathrm{CO}}$, which corresponded to 1.3 mm for t=1  mm and 2.8 mm for t=3  mm. For $\Delta s>\Delta s_{\mathrm{CO}}$, both ${\mathrm{SAM}}_{\text{Meat}}$ and ${\mathrm{SAM}}_{\mathrm{Il}}$ decreased monotonically. However, the slope of decrease of ${\mathrm{SAM}}_{\mathrm{Il}}$ as a function of Δs was larger, showcasing that for large Δs values only the subsurface protein-rich layer could be detected. A similar behavior was observed for the phantom with the smallest Intralipid thickness (t=0.5  mm). However, no crossover point was observed in this case because $\Delta s_{\mathrm{CO}}$ would have occurred at a Δs value smaller than 0.5 mm.

The detectability of the subsurface layer proved more difficult when the top layer was associated with porcine fat. In this case, a crossover point was detected at Δs=4  mm when the adipose-rich layer thickness was 1 mm. Detectability of the subsurface protein-rich layer could not be achieved for larger values of t. The optical properties of the top layer are unknown but are associated with an absorption coefficient higher than that Intralipid, but with a reduced scattering coefficient likely of the same order. The observed behavior—in terms of SAM values as a function of Δs—is consistent with the trends observed for the phantoms C09AD and C10AD, i.e., the phantoms for which the PDMS layer was associated with high absorption and high scattering. For these cases, a crossover point could only be detected for t<2  mm.

## 4 Discussion and Conclusion

We developed a protocol, in tissue phantoms, that allowed the creation of curves predicting sensing depth for a particular spatial offset in spatially offset Raman spectroscopy. The method is applicable for cases when prior information is known relating to the optical properties $\mu_{a}$ and $\mu_{s}^{\prime }$. A set of bilayer PDMS/Nylon tissue phantoms was produced, as well as a proof-of-concept experiment in biological phantoms made of protein-rich tissue and a fatty substance, either Intralipid or porcine fat. Intralipid is a lipid emulsion commonly used in optical phantoms due to its well-characterized scattering properties, which resemble those of human tissue, particularly adipose tissue due to its high lipid content. Although Intralipid does not fully replicate the absorption characteristics of real fat, it was used here as a scattering-mimicking medium. An experiment closer to real-world applications was also performed using porcine fat as the top layer of the tissue phantoms.

The preliminary results of this study indicate that a model-based approach could be developed in which sampling depth is modulated through spatial offset changes, provided the absorption and elastic scattering properties of the sample are known. Within this framework, the fitting parameters A and φ in Eq. (2) should be viewed as empirical constructs that encapsulate the combined influence of these optical properties, rather than as quantities with a straightforward analytical dependence on $\mu_{a}$ and $\mu_{s}^{\prime }$. Any attempt to infer such a dependence directly from our dataset would be speculative, except in trivial edge cases—for instance, when absorption is negligible and depth sensitivity is governed primarily by scattering, or conversely, when scattering is negligible and absorption dominates. A rigorous treatment would instead require Monte Carlo light transport tissue simulations to generate a lookup table covering the relevant range of absorption and scattering coefficients for biological tissues. Importantly, these parameters are not expected to depend on the Raman spectral identity of the layers (e.g., tissue type). The Spectral Angle Mapper (SAM) was employed only as a pragmatic criterion to identify the crossover offset $\Delta s_{\mathrm{CO}}$, where superficial and subsurface contributions balance. Although SAM introduces some approximation error, the fitted parameters remain fundamentally linked to bulk photon transport rather than to specific spectral features. Consequently, practical implementation of the model requires calibration of A and φ for the optical properties of interest, either experimentally, as demonstrated here, or numerically via Monte Carlo–based lookup tables. Once calibrated, Eq. (2) enables prediction of the effective median sampling depth—that is, the depth at which approximately half of the detected signal originates from above and half from below—for a given spatial offset, thereby allowing controlled adjustment of penetration depth or depth-resolved measurements across a range of offsets.

The prediction of the optimal spatial offset in a SORS imaging system to probe a specific depth was done using bilayer solid optical phantoms for various optical property combinations of the top layer. The availability of predictive curves for SORS imaging systems could help develop more robust and standardized depth-resolved Raman spectroscopy approaches in a clinical context. As an example, breast cancer is the most common and deadliest cancer among women worldwide.^43^ Standard treatment procedures for breast cancer include surgical excision of the tumor, followed by radiotherapy of various degrees of aggressiveness. Breast-conserving surgery is usually the preferred surgical approach, but current practices still result in a reoperation rate of 14% to 25%, leading to increased risks of complications and higher costs.^44^^–^^48^ As this procedure implies removing only the tumor from the breast, a challenge lies in the assessment of the surgical margins of the excised tissue. Cancer cells may be left in the patient if the margin is too close to the surface, even if the surface of the excised tumor is associated with normal tissue. Our group has already developed a margins inspection technique using the hyperspectral line-scanning system. However, the depth-resolution potential of the system, based on SORS, had not been considered. In fact, the method was shown to be mostly superficial, being able to achieve no more than half-a-millimeter sensing depth. However, in breast-conserving surgery, there could be cases where tissue remains underneath a layer of fat and may be detectable with SORS. This work preliminarily demonstrated the potential of SORS to detect nonfat tissue under a layer of a fatty substance, opening the door to the development of depth-resolved tumor detection in breast cancer. Future work is needed however to assess the real-world potential of the technique based on more realistic tissue phantoms. Moreover, the impact of interference of the Raman signal from the top layer on predictive tumor detection machine learning models will need to be assessed, as well as the potential impact of the presence of extravasated blood interfering with the Raman tissue signature.

The choice of the SAM metric and the crossover spatial offset to assess depth sensitivity comes from an approximation of the diffusion theory of photon propagation in turbid media. Considering a measured SORS spectrum is a linear combination of both material spectra and ever-present stochastic noise, we assumed that when the SAM metric gives an equal value for both reference spectra, then each material has an equal contribution in the measured signal. This suggests that half of the backscattered photons came from each material and that the overall depth penetration of the banana-shaped diffusion path must be at the interlayer interface. This simplification of the diffusion theory may not represent the true behavior of photons, and a more complex analysis will be needed to predict the depth penetration from spatial offset more precisely. Related to diffusion theory, the choice of the fitted curve equation is not universally accepted, even if experimental data point tends to confirm it. Some PDMS recipes do not fit well with the hypothesized relationship due to the lack of data points, reinforcing the need for a more complete set of optical phantoms.

Although spectral angular mapping has proven effective in our current implementation for decoupling the contributions of two molecular species with distinct spectral signatures, we acknowledge that it represents a relatively simple approach. In future work, we plan to explore more advanced spectral unmixing techniques to enhance robustness and accuracy, particularly in complex or noisy environments. Potential candidates include non-negative matrix factorization (NMF),^49^ independent component analysis (ICA),^50^ multivariate curve resolution (MCR),^51^ and sparse unmixing methods.^52^ These approaches may offer improved performance in resolving overlapping spectra or handling contributions from additional molecular components. However, such analyses lie beyond the scope of the present study and will be addressed in future investigations.

## Data Availability

The data and materials information that support the findings of this study are available from the corresponding author upon reasonable request.
